# Supplementary material for: Persistent dark states in anisotropic central spin models
Source: Sci Rep. 2020 Sep 30;10:16080. doi: 10.1038/s41598-020-73015-1 (PMC7527970; doi:10.1038/s41598-020-73015-1)
Supplement: Supplementary file 1 — Supplementary Information. [file 41598_2020_73015_MOESM1_ESM.pdf]

Supplementary Information: Persistent dark states in anisotropic central spin models

Tamiro Villazon[1,*], **Pieter W. Claeys**[2], **Mohit Pandey**[1], **Anatoli Polkovnikov**[1], **and Anushya Chandran**[1]

[1]Department of Physics, Boston University, 590 Commonwealth Avenue, Boston, Massachusetts 02215, USA
[2]TCM Group, Cavendish Laboratory, University of Cambridge, Cambridge CB3 0HE, UK
[*]rtvs@bu.edu

Energy spectrum and central spin entanglement off resonance. The main text introduces the Hamiltonian with a local magnetic field ω_0 on the central spin, and a global field ω on the bath:

$$H = \omega_0 S_0^z + \omega \sum_{i=1}^{L-1} S_i^z + \sum_{i=1}^{L-1} g_i \left(S_0^x S_i^x + S_0^y S_i^y + \alpha S_0^z S_i^z \right), \tag{S1}$$

where α tunes the qubit-bath interaction anisotropy and g_i describes the qubit-bath interaction strengths $g_i = g_0(1+\gamma\delta_i)$. The total magnetization $\sum_{j=0}^{L-1} S_j^z$ commutes with H, giving rise to polarization sectors with definite total magnetization.

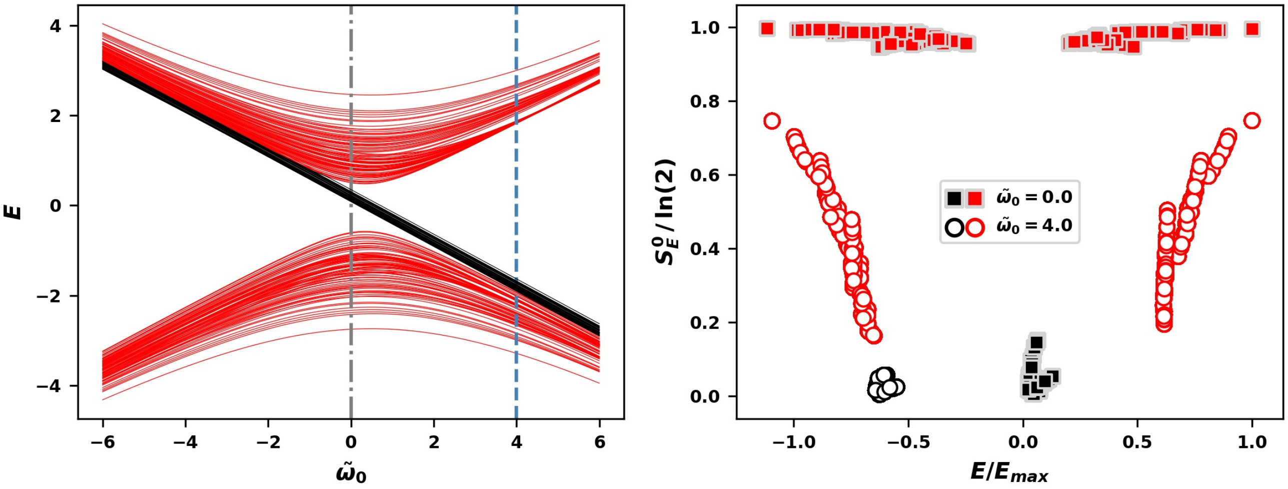

Supplementary Figure S1. Spectrum and central spin entanglement on and off resonance. Left panel plots the energy E as a function of the effective central field $\tilde{\omega}_0$, for all eigenstates of H. Right panel shows the central spin entanglement for all eigenvalues on resonance ($\tilde{\omega}_0 = 0$) and off resonance ($\tilde{\omega}_0 = 4$). Vertical lines in the left panel denote the field values at resonance (gray dash-dotted line) and off resonance (blue dashed) used in the right panel. On resonance, dark and bright states can be easily distinguished by E and S_E^0, while off resonance these observables become comparable. Parameters: $L = 11$, $N_s = 1$ (typical sample), $\alpha = 0.5$, $\gamma = 0.5$, $\sum_j S_j^z = -0.5$.

This model has a natural resonance point at which the effective z-field on the central spin and the surrounding bath spins are equal. At this point, the exchange interactions between the central spin and the bath are strongly enhanced. At $\alpha = 0$, resonance occurs when $\omega_0 = \omega$. At finite $\alpha > 0$ and in a fixed polarization sector, the last term in H shifts the resonance point since $\alpha g_0 S_0^z \sum_{j=1}^{L-1} S_j^z = \alpha g_0 S_0^z (\sum_{j=0}^{L-1} S_j^z - S_0^z) = \alpha g_0 S_0^z \sum_{j=0}^{L-1} S_j^z - h_0$, where $h_0 = \alpha g_0 (S_0^z)^2 = \alpha g_0/4$ is a constant for central spin $1/2$. Collecting the terms in the Hamiltonian coupled to the central spin S_0^z yields the shifted resonance condition:

$$\tilde{\omega}_0 \equiv \omega_0 + \alpha g_0 \sum_{j=0}^{L-1} S_j^z = \omega. \tag{S2}$$

Without loss of generality, we set $\omega = 0$ throughout this work, such that the resonance condition is given by $\tilde{\omega}_0 = 0 \implies \omega_0 = -\alpha\, g_0 \sum_{j=0}^{L-1} S_j^z$.

The results shown in the main text focus on the physics of the system near resonance, where the difference between bright and dark states is most pronounced. This distinction is most clearly seen in the XX limit ($\alpha = 0$), where dark states are product states $|\downarrow\rangle_0 \otimes |\mathcal{D}^-\rangle$ or $|\uparrow\rangle_0 \otimes |\mathcal{D}^+\rangle$, whereas bright states have the form $c_1(\omega_0)\,|\downarrow\rangle_0 \otimes |\mathcal{B}_\downarrow\rangle + c_2(\omega_0)\,|\uparrow\rangle_0 \otimes |\mathcal{B}_\uparrow\rangle$, with nonzero c_1 and c_2 dependent on ω_0. A thorough discussion of the spectrum in the XX limit is given in Ref. 1. Dark states are insensitive to changes in ω_0. In contrast, bright states can be tuned to equal superpositions of the central spin up and down at resonance ($\omega_0 = 0$), or configurations where the central spin is mostly polarized along either direction (as $\omega_0 \to \infty$, $c_1 \to 0, c_2 \to 1$ and as $\omega_0 \to -\infty$, $c_1 \to 1, c_2 \to 0$). Thus the central spin can be essentially decoupled from the bath in bright states with strong off-resonant fields.

Figure S1 shows the energy spectrum of H (left panel) across a range of shifted central field values $\tilde{\omega}_0$, and the central spin entanglement entropy (right panel) for $\tilde{\omega}_0 = 0$ (squares) and $\tilde{\omega}_0 = 4$ (circles) – see vertical dash-dotted and dashed lines in the left panel respectively. We have fixed the total magnetization to be $\sum_{j=0}^{L-1} S_j^z = -0.5 < 0$, such that dark states have $\langle S_0^z \rangle \approx -0.5$. In the spectrum, bright states come in pairs exhibiting level repulsion at resonance (see bands of red curves). Dark states show up as linear bands of near degenerate states (see black lines). Far from resonance, the central spin is nearly polarized in bright eigenstates, and has low entanglement entropy. The distinction between dark and bright states (as measured by observables such as $E, \langle S_0^z \rangle$, and S_E^0) thus becomes progressively less sharp away from resonance, and must be characterized by alternative means (e.g. by their sensitivity to ω_0).

Central spin projection: breakdown of perturbation theory. In the main text, we established how perturbation theory captures the behavior of observables such as the central spin expectation value $\langle \mathcal{D}(\alpha,\gamma)|S_0^z|\mathcal{D}(\alpha,\gamma)\rangle$, for a broad range of anisotropies α and small to moderate disorder γ. When $\gamma \gtrsim 1.0$, perturbation theory breaks down more rapidly as we tune α away from the $\alpha = 0$ integrable line. This is shown in Fig. S2.

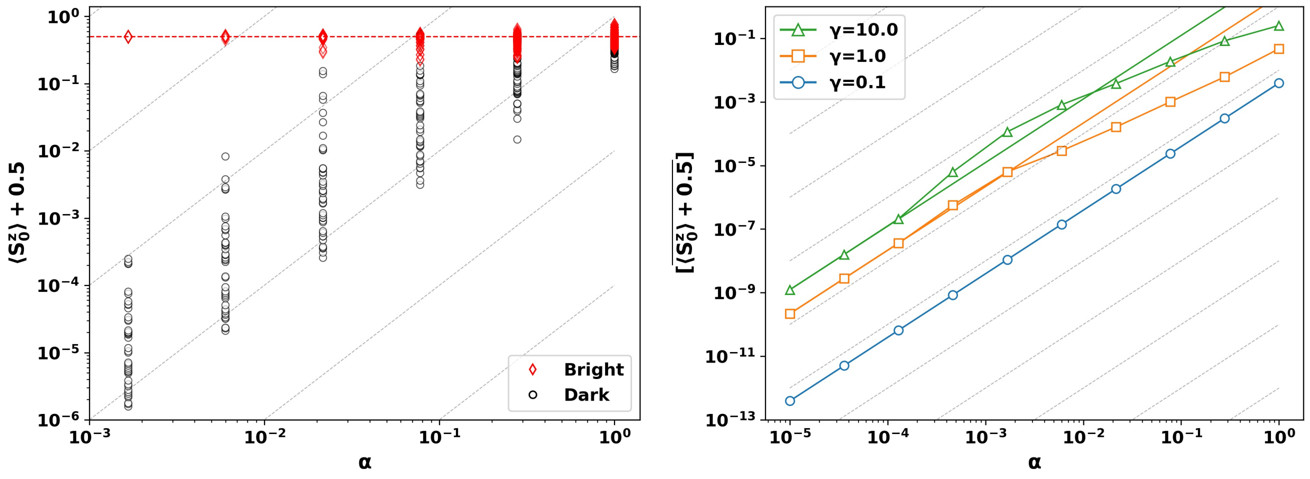

Supplementary Figure S2. Perturbation theory breaks down rapidly at large γ. Left plot shows the eigenstate expectation value of the central spin z-projection $\langle S_0^z \rangle$ for a typical sample of disorder with strength $\gamma = 10.0$. We see deviations from perturbation theory due to mixing between dark and bright states. The color coding used to separate dark and bright states is only nominal at sufficiently large α, as the states can no longer be precisely separated into two distinct clusters. Right plot shows the expectation $\overline{[\langle S_0^z \rangle + 0.5]}$ averaged over the N_D eigenstates with smallest central spin projection, and $N_s = 500$ disorder samples. The numerical data (markers) with $\gamma = 1.0$ and $\gamma = 10.0$ showcase the breakdown of perturbation theory for $\alpha \gtrsim 10^{-4}$ (solid lines). Parameters $L = 12$, $N_s = 500$ (right), $\sum_j S_j^z = -1$, $\omega = \alpha$.

Locality of the adiabatic gauge potential. The adiabatic gauge potential (AGP) $\mathcal{A}_\alpha$ presented in the main text was used to develop a perturbation expansion (Section II), as well as establishing chaos (Section IV). The robustness of perturbation theory in our present context can be traced back to the locality of AGP; that is, $\mathcal{A}_\alpha$ is dominated by few-body terms at mesoscopic system sizes. In the main text, we presented the decomposition:

$$\mathcal{A}_\alpha = \sum_{k=1}^{L} \sum_{\{p_i\}} \sum_{\{\lambda_j\}} J_{\lambda_1,\ldots,\lambda_k}^{p_1,\ldots,p_k}\, \sigma_{p_1}^{\lambda_1} \cdots \sigma_{p_k}^{\lambda_k}, \tag{S3}$$

where $\sigma_{p_i}^{\lambda_j}$ with $\lambda_j \in \{x,y,z\}$ denote the Pauli basis operators on site p_i, where $0 \le p_1 < p_2 < \ldots < p_k \le L-1$ for every $k = 1,\ldots,L$. In principle $\mathcal{A}_\alpha$ has contributions from operators with all possible supports. However, in Fig. S3, we show that $\mathcal{A}_\alpha$ for small $\alpha \ll 1$ has non-zero weight only for k-body operators with $k = 3,5,7,\ldots$, and is dominated by 3-body terms.

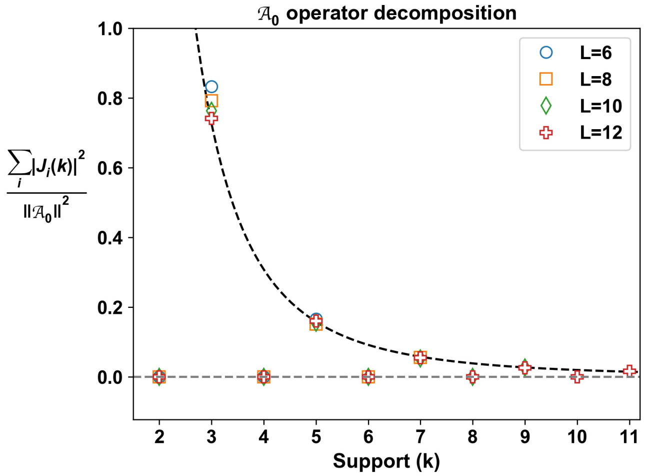

Supplementary Figure S3. Locality of the $\mathcal{A}_\alpha$. The vertical axis of the figure shows the sum of all squared-coefficients for operators with k-body terms (normalized by the trace norm squared of $\mathcal{A}_\alpha$). The horizontal axis gives the support (k). The AGP $\mathcal{A}_\alpha$ has contributions only from operators with odd support. It is dominated by 3-body terms, and exhibits a power law decay $\sim k^{-c}$. The exponent $c \approx 3$ was found by linear regression on a log-log plot. Parameters: $N_s = 1$, $\omega = \alpha$, $\gamma = 0.5$, $\alpha = 0$.

Inverse participation ratio for persistent dark states. In the main text, we characterized persistent dark states based on properties of the central spin in eigenstates. Persistent dark states can also be identified by their inverse participation ratio (IPR) relative to energy eigenbasis as $\alpha \to 0$:

$$\text{IPR} \equiv \sum_n |\langle n(0)|\mathcal{D}(\alpha)\rangle|^4, \tag{S4}$$

where $\{|n(0)\rangle\}$ is the set of eigenstates (bright and dark) at $\alpha \to 0$, and $|\mathcal{D}(\alpha)\rangle$ is any persistent dark state at $\alpha > 0$. As $\alpha \to 0$, the persistent dark state coincides with a single unperturbed dark state; then IPR= 1 and the dark state can be thought of as being “localized" in the reference ($\alpha = 0$) energy basis. We expect that the IPR decreases on increasing α from zero as the perturbed dark state has significant weight on an increasing number of unperturbed eigenstates. The IPR is bounded from below by the value $1/D$, where D is the Hilbert space dimension of the appropriate polarization sector. A perturbed dark state can saturate this bound if it becomes an equal superposition of all reference states; then the dark state can be thought of as being fully “de-localized" in the reference basis.

Figure S4 shows the behavior of the IPR for all persistent dark states over $N_s = 50$ disorder realizations in a single magnetization sector $\sum_j S_j^z = -1$. The figure plots the quantity $(1-\text{IPR})$ against α over two orders of magnitude. The dark unfilled circles show a distribution of IPR values for the different persistent dark states around their respective average values shown as red filled circles. Dark state IPRs collectively decrease with increasing α, with some persistent dark states approaching the bound $1-\text{IPR} = 1-1/D$ (gold dashed line) at the highest α values. Note however that many persistent dark states are robust, showing little mixing and remaining highly localized in the reference basis even at the largest α. The average participation ratio is found to satisfy the scaling:

$$\overline{[1-\text{IPR}]} \sim \alpha^2 \tag{S5}$$

in accordance to perturbation theory (see dotted grey lines with $O(\alpha^2)$ scaling for reference).

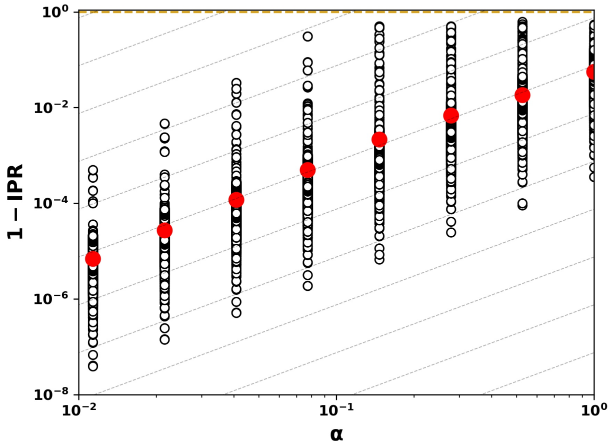

Supplementary Figure S4. Dependence of the dark state inverse participation (IPR) ratio on the anisotropy parameter. Figure shows $1-$IPR vs α for all persistent dark states (black unfilled circles) over $N_s = 50$ disorder samples in the $\sum_j S_j^z = -1$ polarization sector. The solid red circles denote the average over dark states and disorder samples. The gold horizontal dashed line marks the value $1-1/D$ at maximal participation. The reference dotted grey lines have a scaling $\sim \alpha^2$. The average $1-$IPR is found to scale as $\mathcal{O}(\alpha^2)$ in accordance with perturbation theory. Parameters: $L = 12$, $\omega_0 = \alpha$, $\sum_j S_j^z = -1$, $N_s = 50$, and $\gamma = 0.5$.

Adiabatic gauge potential norm for variations in disorder strength. The adiabatic gauge potential which generates translations in γ-space is denoted by $\mathcal{A}_\gamma$. The behavior of $\mathcal{A}_\gamma$ is analogous to $\mathcal{A}_\alpha$, and can be used to study integrability-breaking perturbations, as well as the onset of chaos by tuning γ. Figure S5 shows the exponential divergence of the Frobenius norm of $\mathcal{A}_\gamma$ as a function of system size L.

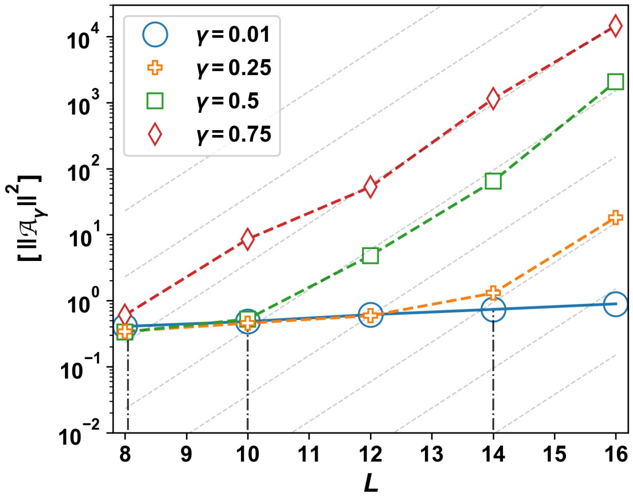

Supplementary Figure S5. Exponential divergence of the of disorder averaged norm $\mathcal{A}_\gamma$ with system size. Close to the integrable point ($\gamma = 0.01$), the norm scales sub-exponentially. The curves for larger γ break off from the $\gamma = 0.01$ line at a critical size L^* and subsequently grow exponentially with slope $2\ln(2)$, reflecting slow relaxation. Parameters: $N_s = 200$, $\alpha = 0.5$, $\omega = \alpha$, $\sum_j S_j^z = -1$, $c \approx 1$.

Persistent dark states and adiabatic gauge potential norm scaling in different magnetization sectors. In the main text, our numerical results focused on the magnetization sector $\sum_j S_j^z = -1$ with zero magnetization density $\lim_{L\to\infty} \sum_j S_j^z/L \to 0$. This sector is the largest one containing both dark and bright states (note the $\sum_j S_j^z = 0$ sector contains only bright states). In this section, we present analogous results (specifically analogs of Figures 3 and 8 in the main text) in a magnetization sector with non-zero density $\sum_j S_j^z/L = -1/4$. As the Hilbert space dimension of this sector is smaller than that of the $|\sum_j S_j^z| = 1$ sector, our numerical simulations probe larger system sizes $L \approx 20$.

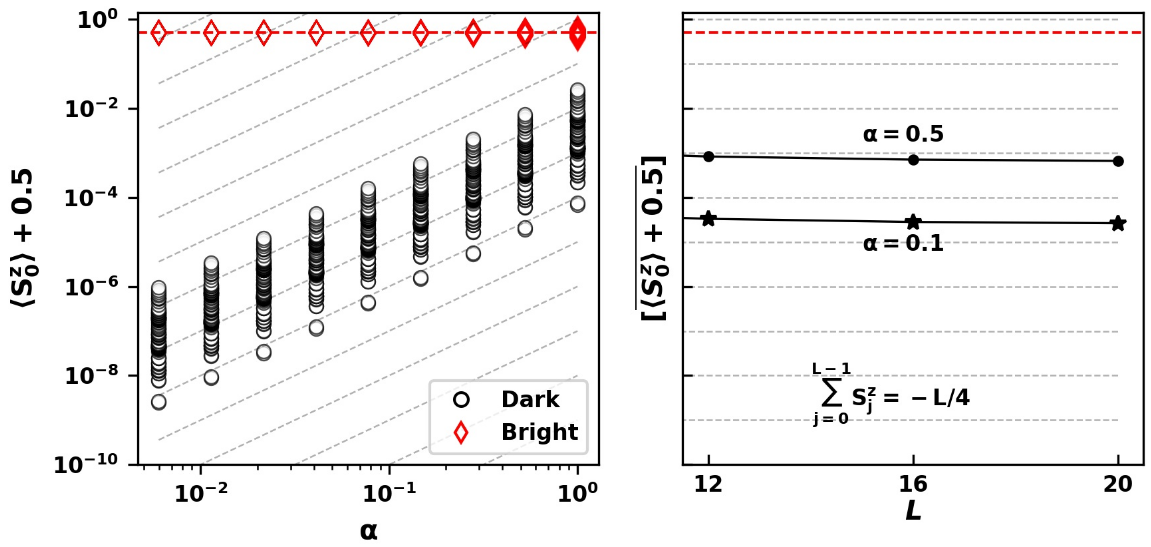

Supplementary Figure S6. Dark states persist away from the integrable lines at finite size at non-zero magnetization density . Left panel: Expectation value of the central spin z-projection for every eigenstate of H in a typical sample as a function of α. Persistent dark (black circles) and bright (red diamonds) states are easily distinguished by their values of $\langle S_0^z \rangle$. Dotted lines (gray) show α^2 scaling, while the horizontal dashed line (red) indicates $\langle S_0^z \rangle = 0$. Right panel: System size dependence of the expectation value $\overline{[\langle S_0^z \rangle + 0.5]}$ on averaging over N_s disorder samples and the N_D eigenstates with smallest z-projection. Parameters: $L = 12$ (left), $N_s = 500$ (right), $\omega_0 = L\alpha/4$, $\sum_j S_j^z = -L/4$, and $\gamma = 0.5$.

Figure S6 extends the results of Figure 3 of the main text to the $\sum_j S_j^z = -L/4$ sector. The left panel shows the expectation value of the central spin z-projection for all eigenstates of H as a function of the anisotropy parameters α. The result is qualitatively similar to the result in the $\sum_j S_j^z = -1$ sector: dark states persist away from the integrable line, are clearly distinguishable from perturbed bright states, and their central spin expectation value scales in accordance with perturbation theory as $O(\alpha^2)$. The right panel shows the average of the central spin z-projection over all persistent dark states and disorder samples plotted against system size L. Up to the largest accessible system size ($L \approx 20$), the averaged central spin z-projection remains approximately constant. Thus dark states also persist at the largest accessible sizes in magnetization sectors with non-zero density.

Figure S7 extends the results of Figure 8 of the main text to the $\sum_j S_j^z = -L/4$ sector. The figure shows the adiabatic gauge potential norm as a function of system size L for several values of the anisotropy parameter α. As in the main text, the gauge potential norm is a polynomial of L in accordance with an integrable perturbation of an integrable system up to a critical system size L^*. For $L > L^*$, the AGP norm grows exponentially with L:

$$||\mathcal{A}_\alpha||^2 \sim 2^{2L}. \tag{S6}$$

The inset shows that the critical size L^* is linearly dependent on $\log_2(\alpha)$, just as in the inset of Figure 8 in the main text. The slope $\nu \approx 2.5$ is however twice as large as the value in the $\sum_j S_j^z = -1$ sector. These results are consistent with an exponentially diverging relaxation time $\tau_r \sim C|\alpha|^{2\nu} 2^L$. Therefore, the system size scaling of the gauge norm and the associated relaxation time continues to hold in magnetization sectors with non-zero density.

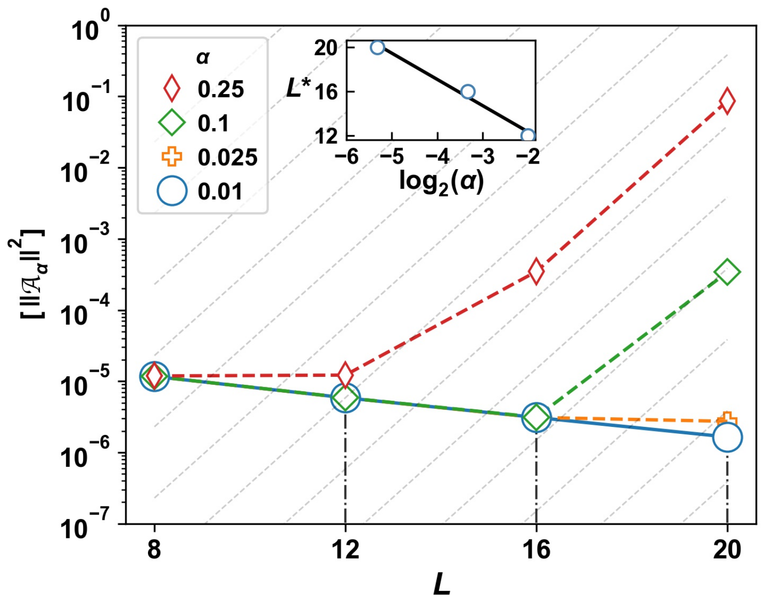

Supplementary Figure S7. The exponential divergence of the adiabatic gauge potential norm shows signatures of chaos in sectors with non-zero magnetization density. Plot shows the disorder-averaged norm $[\|\mathcal{A}_\alpha\|^2]$ as a function of system size L. Dotted lines show the scaling behavior in the chaotic non-ergodic regime $[\|\mathcal{A}_\alpha\|^2] \sim 2^{2L}$. Vertical dashed-dot lines mark the onset of exponential growth at $L^*(\alpha)$. Inset: $L^*(\alpha)$ vs. $\log_2(\alpha)$, and a regression line whose slope is numerically found to be $-\nu \approx -2.5$. Parameters: $\gamma = 0.5$, $N_s = 200$, $\omega_0 = L\alpha/4$, $\sum_j S_j^z = -L/4$, $\mu/L = 2^{-L}/c$, with $c \approx 10$.

Resonant energy gap and dark-bright hybridization. In the main text, we discussed various contributions to the AGP norm in the chaotic non-ergodic regime (Figure 10). In particular, we found that the contribution from dark-bright mixing (DB) increased exponentially with L only at large α ($\alpha = 0.5$). Here, we correlate this rise with a closing of a finite-size energy gap between the dark and bright manifolds in the spectrum.

Figure S8 shows the distribution of dark and bright energies as a function of system size L, for $\alpha = 0.1$ and 0.5. At $\alpha = 0.1$ (left panel), the dark and bright manifolds are separated by an energy gap at the accessible system sizes, so that the dark and bright states only weakly hybridize. This explains the lack of exponential growth with L of the DB component of the AGP norm in the left panel of Figure 10 of the main text. On the other hand, at $\alpha = 0.5$ (right panel), the dark and bright manifolds overlap in energy for $L \geq 14$ and can strongly hybridize. This strong hybridization results in the exponential rise of the DB component of the AGP norm for $L \approx 12$ in the right panel of Figure 10 in the main text.

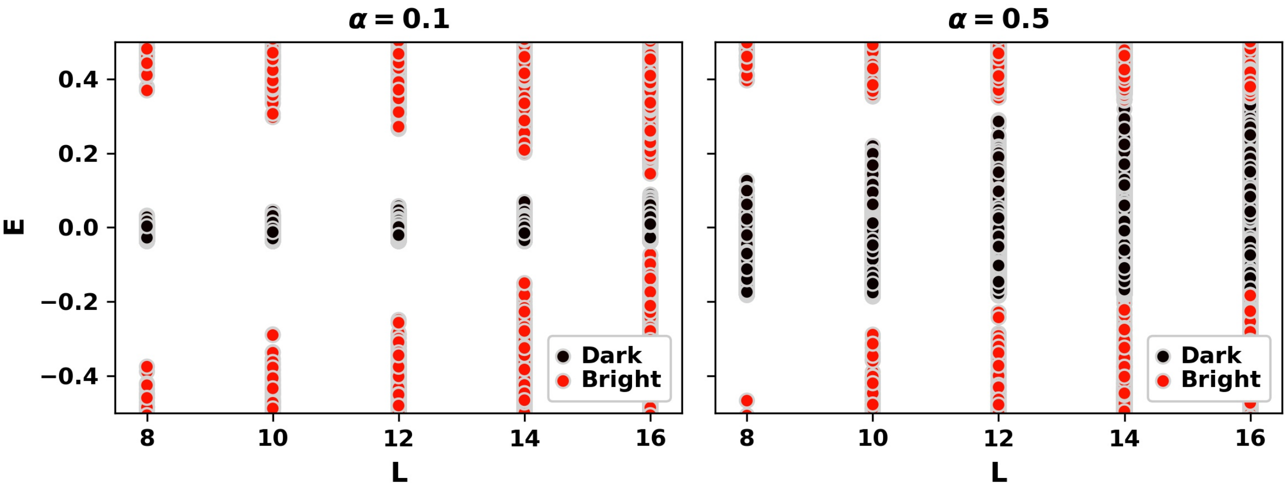

Supplementary Figure S8. Dark-bright energy hybridization with system size. Plot shows the distribution of dark and bright energies as a function of system size L in the $\sum_j S_j^z = -1$ magnetization sector. We show two values of anisotropy, $\alpha = 0.1$ (left panel) and $\alpha = 0.5$ (right panel) in accordance with Figure 10 of the main text. The bright-dark gap remains open at all accessible system sizes for $\alpha = 0.1$, while the gap closes by $L = 14$ for $\alpha = 0.5$. Parameters: $\gamma = 0.5$, $\omega_0 = \alpha$, $\sum_j S_j^z = -1$, and $N_s = 100$.

References

1. Villazon, T., Chandran, A. & Claeys, P. W. Integrability and dark states in an anisotropic central spin model. *arXiv preprint arXiv:2001.10008* (2020).
